# Adipose Tissue in Breast Cancer Microphysiological Models to Capture Human Diversity in Preclinical Models

**DOI:** 10.3390/ijms25052728

**Published:** 2024-02-27

**Authors:** Katie M. Hamel, Trivia P. Frazier, Christopher Williams, Tamika Duplessis, Brian G. Rowan, Jeffrey M. Gimble, Cecilia G. Sanchez

**Affiliations:** 1 Obatala Sciences, Inc., New Orleans, LA 70148, USA; katie.hamel@obatalasciences.com (K.M.H.); trivia.frazier@obatalasciences.com (T.P.F.); jeffrey.gimble@obatalasciences.com (J.M.G.); 2 Division of Basic Pharmaceutical Sciences, Xavier University of Louisiana, New Orleans, LA 70125, USA; cwilli35@xula.edu; 3 Delgado Community College, New Orleans, LA 70119, USA; tduple@dcc.edu; 4 Department of Structural and Cellular Biology, Tulane University School of Medicine, New Orleans, LA 70112, USA; browan@tulane.edu

**Keywords:** breast cancer, microphysiological system, 3D culture, diversity, adipose tissue, adipose-derived stromal/stem cells, adipocytes, tumor microenvironment, tumor stroma

## Abstract

Female breast cancer accounts for 15.2% of all new cancer cases in the United States, with a continuing increase in incidence despite efforts to discover new targeted therapies. With an approximate failure rate of 85% for therapies in the early phases of clinical trials, there is a need for more translatable, new preclinical in vitro models that include cellular heterogeneity, extracellular matrix, and human-derived biomaterials. Specifically, adipose tissue and its resident cell populations have been identified as necessary attributes for current preclinical models. Adipose-derived stromal/stem cells (ASCs) and mature adipocytes are a normal part of the breast tissue composition and not only contribute to normal breast physiology but also play a significant role in breast cancer pathophysiology. Given the recognized pro-tumorigenic role of adipocytes in tumor progression, there remains a need to enhance the complexity of current models and account for the contribution of the components that exist within the adipose stromal environment to breast tumorigenesis. This review article captures the current landscape of preclinical breast cancer models with a focus on breast cancer microphysiological system (MPS) models and their counterpart patient-derived xenograft (PDX) models to capture patient diversity as they relate to adipose tissue.

## 1. Introduction

Female breast cancer represents approximately 15.2% of all new cancer cases in the United States, with an estimated 297,790 new cases and 43,170 deaths in 2023 alone [1]. More importantly, data have evidenced increases in breast cancer incidence to 16.9 per 100,000 women, while efforts in drug development lead to limited efficacy. Approximately 85% of the drugs in early clinical trials fail due to poor clinical efficacy, toxicity, and drug-like properties [2,3]. These roadblocks to clinical translation have initiated a shift in the reexamination of preclinical models used for breast cancer drug development. Classic animal models still serve as the gold standard for disease modeling and compound validation.

Observable variance in breast cancer incidence, mortality, treatment response, and recurrence across populations has incentivized the development of more clinically relevant in vitro and in vivo models that capture the heterogeneity of the disease [4,5,6,7,8]. In particular, the integration of microphysiological systems (MPS) as humanized three-dimensional models has opened new avenues and opportunities for precision medicine in the treatment of breast cancer. Preclinical models range from traditional, static, homotypic culture models to MPS models that include engineered organoids, single organ/tissue chips, and multi-organ interconnected models [9]. In addition, other physiologically relevant in vivo models that allow the study of diverse patient responses, including the chorioallantoic membrane (CAM) and patient-derived xenograft (PDX) models for drug development and disease modeling, are being adopted as patient-specific tumor therapy models [10,11,12]. The ideal attributes of breast cancer MPS models, as emphasized by Bissell, Griffith, Prestwich, and colleagues, include the use of human-derived materials, extracellular matrix (ECM), and a heterogeneous cell population, amongst other design criteria [13]. These properties support the goal of recapitulating breast inter- and intra-tumoral heterogeneity by integrating patient-derived cells and mimicking the breast tumor microenvironment (TME) observed in vivo. Nevertheless, given the anatomical location of mammary adipose tissue and the documented pro-tumorigenic role of adipocytes in both early and late stages of tumor progression, there remains a need to enhance the complexity of current models and account for the contribution of the components that exist within the adipose stromal environment on breast cancer initiation, progression, invasion, and treatment response. Here we present an overview of breast cancer-adipose MPS models and discuss their feasibility in mimicking human and tissue diversity. 

### 1.1. Diversity in Breast Cancer 

**Figure 1:**
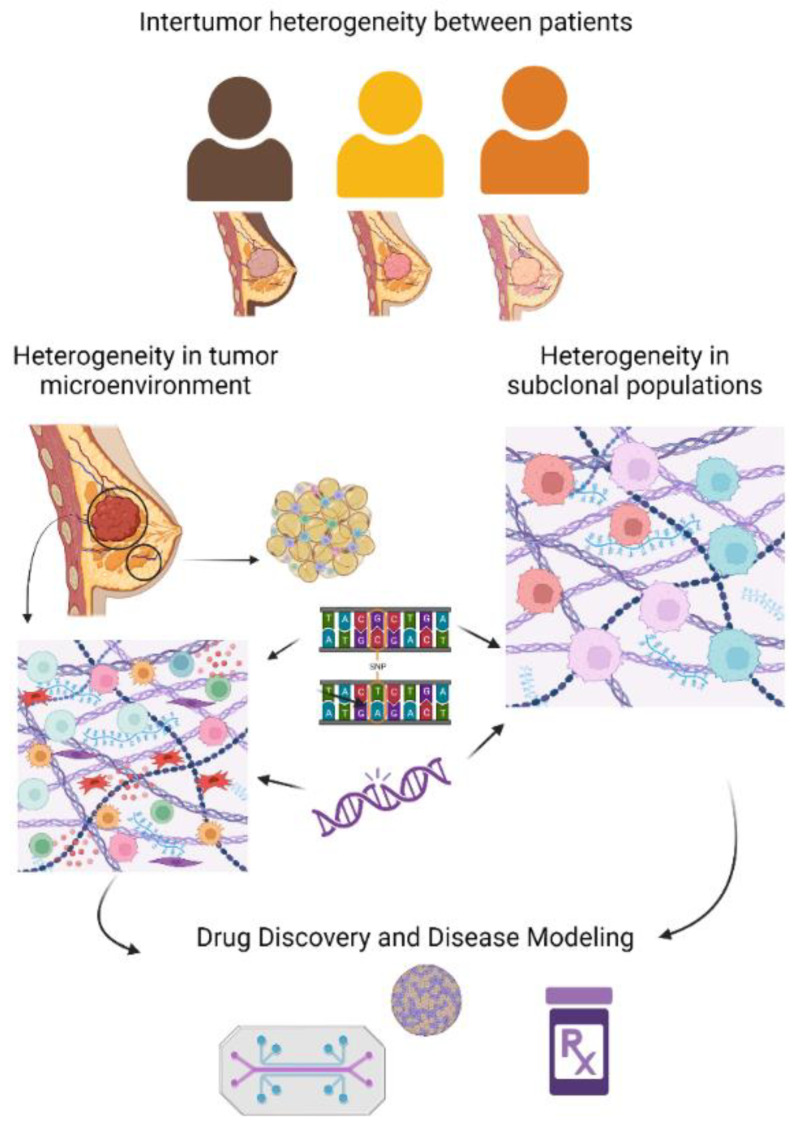
Tumor heterogeneity contributes to therapeutic resistance, resulting in tumor recurrence and metastasis. Tumor heterogeneity is divided into two subcategories: inter- and intra-tumor heterogeneity. Patient-specific differences (inter-tumor) and variations within the tumor itself (intra-tumor) play a critical role in the patient response to treatment. The basis of heterogeneity arises from alterations in the genome (point mutations, single nucleotide polymorphisms (SNPs), insertions/deletions), transcriptome/proteome (over or under expression of genes, variations in patterns of gene expression), and epigenome (variations in DNA methylation and noncoding RNA regulation patterns, differences in chromatin and histone structure). These levels of heterogeneity are substantiated in the tumor microenvironment (TME), expressed biomarkers, metabolic profile, cell cycle, epithelial–mesenchymal transition (EMT), microcirculation of tumor cells, and presenting clinical pathology. Both concepts of intra- and inter-tumoral heterogeneity must be incorporated into current breast cancer microphysiological system (MPS) models to capture the full landscape of the disease and develop more targeted therapies. Created with Biorender.com (accessed on 5 October 2023).

**Figure 2:**
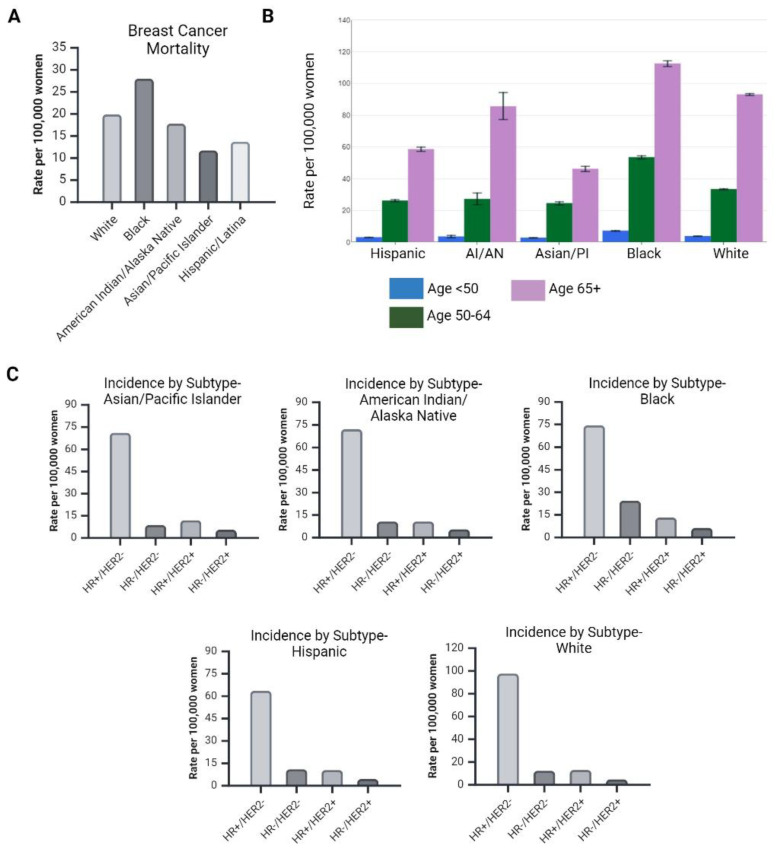
Breast cancer incidence and mortality variance are dependent on race and age. (**A**) Breast cancer mortality in U.S. women by race and ethnicity based on SEER Cancer Statistics Review, 2000–2020, 2022. (**B**) U.S. 5-Year Age-Adjusted Mortality Rates, 2016–2020 per 100,000 women, are adjusted to the 2000 U.S. Std Population. (**C**) U.S. 5-Year Age Adjusted Incidence Rates, 2016–2020 per 100,000 women, are adjusted to the 2000 U.S. Std Population. Created with Biorender.com (accessed on 13 November 2023).

There is heterogenicity in breast cancer disease presentation, incidence, mortality, and treatment response (Figure 1 and Figure 2). Factors attributing to this variance include, but are not limited to, age, race, and ethnicity. For example, breast cancer is the leading cause of cancer deaths among African American and Hispanic women. Furthermore, African American, Hispanic, and American Indian/Alaska Native (AIAN) women are less likely to be diagnosed with local-stage breast cancers compared to Asian/Pacific Islander (API) and White women [5]. African American women are twice as likely to be diagnosed with HR^−^/HER2^−^ tumors compared to Hispanic, AIAN, API, and White women [5]. To factor in age, African American women have the highest incidence rate for <40 years old and the highest mortality rate for ages 20–49 years old due to ER^−^,PR^−^,HER2/neu^−^ (triple-negative, TNBC) breast cancer incidence [5]. White women have the highest incidence at ages 45–49, whereas AIAN women have the highest mortality rate at ages 70–75 [5]. 

Adipose-derived stromal/stem cells (ASCs) and mature adipocytes are a normal part of the breast tissue composition and provide energy, hormonal regulation, and cytokines for wound healing [14]. They not only contribute to normal breast physiology but also play a significant role in breast cancer pathophysiology. ASCs impact tumorigenesis by not only orchestrating ASC recruitment to the tumor site but also by secreting paracrine factors that interact with the tumor stroma. Obesity-induced alterations in adipose tissue also contribute to changes in the biological properties of ASCs, which further enhance tumorigenesis and cancer cell metastasis [15]. The rise in prevalence of obesity and the correlation between obesity and breast cancer mortality, morbidity, and survival have stimulated inquiry into adipose-breast cancer crosstalk as it relates to race, ethnicity, and age [15,16,17]. Although there is strong correlative and causative evidence indicating that obesity impacts ASC and adipocyte function and supports the onset and progression of breast cancer, the extent to which it is linked to race is not well understood. Importantly, the breast TME composition and corresponding biomechanical properties (e.g., tissue stiffness, density) differ between races, ethnicities, and age cohorts [18]. The unique compositional differences in the TME have been demonstrated by profiling resident cell populations, including adipocytes and ASCs, the immune cell landscape, vascular tissue, and the extracellular matrix [19]. 

There are race disparities in obesity and triple negative breast cancer (TNBC) risk. Nevertheless, there is no statistically significant evidence directly linking TNBC to obesity in African Americans or other racial/ethnic demographics [20]. This outcome can be substantiated by the inter-individual variety and extent of obesity-associated medical comorbidities. Furthermore, excessive adipose tissue does not necessarily translate to metabolic dysfunction, as up to thirty percent of obese individuals retain normal indicators of metabolic health [21]. There is also diversity within the adipose tissue, as evidenced by the observation that total body fat, visceral adipose tissue (VAT), subcutaneous adipose tissue (SAT), and body mass index (BMI) are significantly different between varying ethnic and racial backgrounds [22]. These differences could speak to potential variance in ASC stemness, but further comparative studies must be conducted that include ASCs from donors with differing ethnic backgrounds. Although the complex, heterogeneous nature of the breast cancer microenvironment is widely recognized as a critical factor, the application of the knowledge presented in this section as it pertains to preclinical models remains challenging and requires further patient stratification.

### 1.2. Current Breast Cancer-Adipose Preclinical Models 

**Figure 3:**
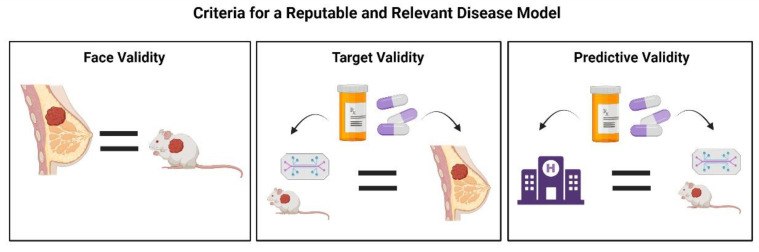
Breast cancer preclinical models must meet three criteria to be considered both reputable and relevant. The translatability of preclinical models plays a decisive role in drug attrition and success rates. For data retrieved from preclinical studies to reflect clinical outcomes, it is important that in vitro disease models meet three criteria: face validity, target validity, and predictive validity. Face validity refers to the biological similarities between the preclinical model and the disease itself. Target validity is attained when drug responses are similar in both the model and clinical setting. Finally, predictive validity is determined based on similar responses observed in both the model and clinical settings. Created with Biorender.com (accessed on 11 January 2024).

**Figure 4:**
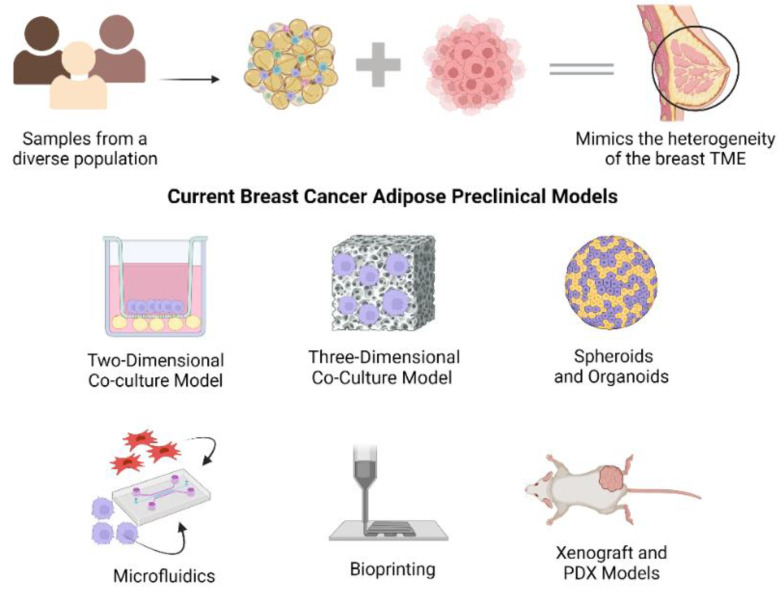
Breast cancer MPS models must reflect patient-specific tumor heterogeneity by incorporating patient-derived tumor cells and associated stroma. Patient demographics, including sex, race, BMI, and health status, are driving factors in breast cancer etiology, progression, and response to treatment. As residents of the breast tissue stroma, adipocytes and ASCs also impact both the normal breast physiology and the pathology attributed to cancer development. Current preclinical models include 2D and 3D co-culture systems and spheroids and organoids, as well as more complex systems including microfluidic devices and bioprinted microenvironments. The engraftment of human breast cancer and adipose-derived cells and tissues in murine models in patient-derived xenograft (PDX) models has become a widely accepted model due to its clinical relevancy. Created with Biorender.com (accessed on 19 October 2023).

The paradigm of in vitro models has shifted from traditional, two-dimensional cell culture to more complex, three-dimensional frameworks, specifically regarding breast cancer and adipose tissue in vitro models. Models strive to capture the heterogeneity associated with the breast cancer tumor microenvironment with the inclusion of multiple cell types and extracellular matrix while also focusing on the temporally dynamic nature of the breast cancer TME. Rezaee et al. emphasized three important criteria for a reputable and relevant disease model: (1) face validity described as biological similarity between the human disease and animal model; (2) target validity, in which the target agents should exhibit similar functions in the model and clinical setting; and (3) predictive validity, in which clinically effective agents function similarly in the disease model [23]. (Figure 3). These criteria, in conjunction with the design criteria described by Bissell et al., represent the ultimate objective for researchers in the development of preclinical breast cancer-adipose tissue models [13,23]. This review article highlights in vitro models established over the past decade, with special attention to those integrating both breast cancer and adipose stroma. These preclinical models include two-dimensional co-culture, three-dimensional co-culture, spheroids, microfluidic devices, and bioprinting applications. Some studies emphasized in this review employ traditional co-culture of ASCs and breast cancer cells in the transwell system [24,25,26,27]. Others introduced a static, three-dimensional microenvironment integrated with the classic co-culture models [28,29]. Several studies established more dynamic co-culture systems [30,31,32]. Finally, whole white adipose tissue was co-cultured with breast cancer cells to create a physiologically relevant system [33]. In addition to these in vitro systems, we also highlight the application of PDX models as a translationally relevant tool to capture patient diversity and recapitulate the role of adipose stroma in breast cancer progression and treatment response (Figure 4). 

#### 1.2.1. Co-Culture Models

Co-culture models investigate the interactions between one or more cell populations, either directly through physical contact via surface receptors and gap junctions or indirectly via cell signaling communication [34]. The latter typically separates the cell types using a semi-permeable membrane, such as a trans-well system. Indirect co-cultures include conditioned media, which contain the cell-derived secretome responsible for positively or negatively regulating cellular behavior via the regulation of signaling pathways. Furthermore, scaffolds are integrated into the co-culture systems to simulate the three-dimensional microenvironment observed in vivo. Hydrogels are the most commonly used scaffolds, with the most prevalent being Matrigel (Corning), an Engelbreth–Holm–Swarm mouse sarcoma-derived basement membrane. Although Matrigel provides additional complexity and relevancy to the model by facilitating cell–matrix interactions, it presents documented drawbacks, including batch-to-batch variability and animal derivation, which impact the face, target and predictive validity metrics described above, and overall clinical relevancy of the model of interest [35].

The use of a co-culture model is critical in the study of adipose tissue as it relates to normal breast tissue and the TME [21,36,37,38,39,40]. The adipose tissue microenvironment can dysregulate normal mammary myoepithelial cell behavior and facilitate breast tumor progression via disruption of routine ECM maintenance and upregulation of adipokines and pro-inflammatory cytokines [24]. Adipocyte paracrine activity can promote breast cancer cell migration, an in vitro hallmark of metastasis [21,28,41,42]. The secretion of adipokines, including adiponectin and leptin, influences breast cancer cell proliferation and invasion. Moreover, IL-6 and leptin secreted by adipocytes lead to subsequent activation of pathways such as JAK/STAT3 and PI3K/AKT, and downstream expression of pro-inflammatory cytokines including Interleukin 6 (IL-6), Interleukin 1 beta (IL-1β), and Tumor necrosis factor alpha (TNF-α) in breast cancer cells [43,44]. Secretion of free fatty acids as well as cytokines and growth factors, including Monocyte chemoattractant protein 1 (MCP-1), C-C motif chemokine ligand 5 (CCL5), and Insulin-like growth factor 1 (IGF-1), also promote breast cancer cell invasion and proliferation [29,45,46,47]. Adipocyte-immune cell interactions play a critical role in breast cancer cell-adipocyte crosstalk and subsequent tumor progression and metastasis. Several studies have demonstrated that macrophages directly (via cell–cell contact) and indirectly (via their secretome) impact adipocyte-breast cancer crosstalk, facilitating tumor angiogenesis and metastasis [27,48]. Although Yadav et al. did not directly implement a breast cancer cell/adipocyte/macrophage co-culture, their co-culture with adipocytes and macrophages was used as a tool to demonstrate a possible mechanism for the increased risk of breast cancer progression observed in obese individuals. 

Other models have incorporated murine-derived or human-derived ASCs or adipocytes with established hormone receptor-positive (HR+) and TNBC cell lines, including MCF-7, MDA-MB-231, T47D, BT-474, and ZR75 cell lines [24,25,26,33,49]. The co-culture models highlighted in Table 1 incorporated adipocytes, or ASCs, derived from a heterogeneous patient population [24,26,33,49]. The experimental design by Ejaz et al. went beyond the traditional 2D co-culture by integrating two unique co-culture methods, including an inverted flask culture, described as a contact co-culture, and a conventional flask culture, identified as a paracrine co-culture [33]. The inverted flask culture included a layer of BT-474, MDA-MB-231, or MCF-7 breast cancer cell lines seeded at the base of the flask, followed by a layer of lipoaspirate derived from female donors undergoing routine abdominoplasty (*n* = 5, 39 ± 13 YO, BMI 27 ± 4 kg/m^2^). The flask remained inverted throughout the cultural period. In contrast, BT-474, MDA-MB-231, and MCF-7 were seeded at the base of a flask, followed by a layer of lipoaspirate collected from the donor pool. The flask was placed in an upright position, allowing the lipoaspirate to float to the top of the flask and create a paracrine co-culture environment. In this study, a traditional trans-well culture with breast cancer cell lines within the well and human ASCs in the insert was used as a comparison. Interestingly, both lipoaspirate and ASCs had no sizeable effect on breast cancer cell proliferation and EMT-related gene expression in either direct or paracrine-dependent co-cultures [33]. 

Co-culture models have improved significantly with the recognition of cell–matrix interactions as a critical driver of cell behavior and the introduction of Matrigel over four decades ago. The incorporation of a matrix not only allows for the use of a heterogeneous cell population, but also facilitates ECM crosstalk [50]. The integration of ECM into in vitro models mimics in vivo cell–matrix interactions, including the conversion of mechanical response ECM properties to a biochemical signal (mechanotransduction) and cell exposure to ECM-embedded chemical signals that include structural proteins and growth factors [51]. Furthermore, evaluation of ECM remodeling via cell-secreted matrix metalloproteases (MMPs) and tissue inhibitors of matrix metalloproteases (TIMPs) can be supported by these types of models [52]. Some adipose-breast cancer models optimized the traditional co-culture transwell model by including a Matrigel coating on the porous membrane or as a third layer sandwiched between multicell layers to evaluate the role of adipose-derived cells in breast cancer cell invasion (Table 1) [28,29,53]. Of these studies, only one included stromal vascular fraction (SVF) and ASCs from multiple patients to assess MDA-MB-231 invasiveness [39]. The other platforms either included 3T3-L1 preadipocytes [28,53] or murine preadipocyte line 3T3-F442A [45]. As an alternative to trans-well co-culture, Asante et al. embedded murine per-uterine and inguinal white adipose tissue (WAT) in Matrigel to collect adipose-derived conditioned media and assess the paracrine effects of lean and obese adipose tissue on MDA-MB-231 mesenchymal–epithelial transition (MET) [54]. Additional scaffolds, including silk, fibrin, collagen, and decellularized matrices, were used as a trans-well coating or more complex in vitro system to evaluate the role of breast cancer-adipocyte crosstalk on breast cancer progression and invasiveness [55,56,57,58]. While many of the three-dimensional co-culture models include human-derived adipocytes, or ASCs, they do not include more than a single donor with undisclosed patient demographics [55,57,59]. A sandwich model is also used to support and evaluate adipocyte-breast cancer cell crosstalk. In this review, we have highlighted the work of Au-Brown, including a combination of non-diseased breast tissue sandwiched between WAT, while Matossian introduces TU-BcX-4IC PDX-derived cells into a modified version of the sandwich white adipose tissue (SWAT) [59,60,61].

**Table 1:** Review of in vitro breast cancer-adipose preclinical platforms, which includes a summary of associated cell types, derivation, key findings, and the strengths and/or weaknesses of the platform demonstrated in each study.

Platform	Cell Type(s)	Human-Derived (Y/N)	Patient-Derived Adipocytes or Adipose-Derived Stromal/Stem Cells (ASCs) (Y/N)	Multi Patient Adipocytes or ASCs (Y/N)	Key Findings	Strengths and/or Weaknesses	Reference
**2D Co-Culture**	THP-1 Macrophages, HUVECs, and Mammary Preadipocytes	Yes	Yes	No	Macrophage vascular endothelial growth factor A (VEGFA) expression increased post co-culture. Macrophage expression of pro-angiogenic and pro-metastatic genes significantly increased post co-culture. Conditioned media derived from co-cultures promoted HUVEC endothelial tube formation	No breast cancer cells were used	[48]
	MSCs, U937, MCF-7, and MDA-MB-231	Yes	Yes	No	Macrophage paracrine activity intensifies breast cancer cell-adipocyte crosstalk	2D co-culture	[27]
	ASCs, MCF-7, T47D, and ZR75	Yes	Yes	Yes	Obesity-altered ASCs contribute to the radiation resistance observed in ER^+^ breast cancer	2D co-culture	[49]
	ASCs, MCF-7, and MDA-MB-231	Yes	Yes	Yes	ASCs increase breast cancer cell proliferation. ASC paracrine activity increases breast tumor cell proliferation	2D co-culture	[26]
	MDA-MB-231 and Adipocytes	No	Yes	No	MDA-MB-231 pro-inflammatory gene expression was upregulated in the presence of obese murine adipose tissue. MDA-MB-231 impacted adipocyte biosynthesis pathways	2D co-culture	[25]
	HS578Bst and ASCs	Yes	Yes	Yes	ASCs disrupted the expression of ECM maintenance-related genes and increased leptin and inflammatory marker gene expression	2D co-culture	[24]
	ASCs, MDA-MB-231, and MCF-7	Yes	Yes	Yes	ASCs do not impact breast cancer cell proliferation via direct cell contact or paracrine activity. ASCs do not significantly increase breast cancer cell EMT-related gene expression via direct cell contact	2D co-culture	[33]
**3D Co-Culture**	3T3-F442A, ZR75, SUM159PT, MCF-7, T47D, and MDA-MB-231	Yes-Breast Cancer Cell Lines No-Preadipocytes	No	No	Adipocytes promote the invasion of MDA-MB-231, MCF-7, and other breast cancer cell lines	No human-derived preadipocytes used	[45]
	ASCs and breast cancer cell lines	Yes	Yes	No	Demonstrates breast cancer cell-primary preadipocyte crosstalk in vitro	Sandwich white adipose tissue-breast cancer model	[59]
	Breast Adipose Tissue, ASCs, and TU-BcX-41C	Yes	Yes	Yes	Increase in cancer stem cell population when TU-BcX-41C cells are cultured in breast cancer-adipose MPS	Modified sandwich MPS model	[60]
	3T3-L1 pre-adipocytes, MDA-MB-231, MCF-7, SUM159, and HS578t	Yes	No	No	Breast cancer cell interactions with the ECM and adipocytes alter breast cancer cell MET, potentially contributing to secondary tumor formation	Used 3T3-L1 preadipocytes	[53]
	MSCs and MDA-MB-231	Yes	Yes	No	MDA-MB-231 invasion is enhanced in the presence of adipocytes and collagen matrix	Integrated a collagen plug into traditional Boyden chamber	[55]
	3T3-L1 pre-adipocytes, MDA-MB-453, MDA-MB-435S, MDA-MB-231, and MDA-MB-468	Yes	No	No	Adipocytes induce migration and invasion of breast cancer cells. Adipocytes stimulate breast cancer cells to adopt an aggressive tumor phenotype by inducing EMT-associated traits	Used 3T3-L1 preadipocytes	[28]
	Stromal vascular fraction (SVF), ASCs, and MDA-MB-231	Yes	Yes	Yes	Direct and indirect contact with adipocytes induces similar invasive behaviors in the MDA-MB-231 TNBC cell line	Enhanced cellular heterogeneity by including SVF	[29]
	Mammary adipocytes and MDA-MB-231	Yes	Yes	Yes	Breast cancer-adipocyte crosstalk is amplified by obesity. Supports the study of mammary adipocyte lipid secretion on tumor secretions and overall tumor aggressiveness in lean and obese conditions	Used a fibrin matrix in Boyden chamber	[56]
	ASCs, Fibroblasts, and MDA-MB-231	Yes	No	No	Collagen VI, a highly oncogenic collagen isoform linked to breast cancer, was decreased in the irradiated cancer co-culture. Irradiation not only makes cells ablative but also may influence the oncogenic potential of the microenvironment	Used decellularized scaffold	[57]
	Murine peri-uterine and inguinal white adipose tissue (WAT) and MDA-MB-231	Yes	No	No	Adipose tissue paracrine activity induces MET-like changes in the MDA-MB-231 TNBC cell line	Used murine WAT	[54]
**Microfluidics**	ASCs and MDA-MB-231	Yes	Yes	No	Statistically significant increase in MDA-MB-231 proliferation in the presence of ASCs. MDA-MB-231 cells adopt more of a mesenchymal phenotype in the presence of ASCs. Paclitaxel has reduced effectiveness in inhibiting MDA-MB-231 replication in the presence of ASCs	One ASC donor used (female, Caucasian, normal BMI)	[30]
	MCF-7 and ASCs	Yes	Yes	Yes	Predicts anastrazole sensitivity with respect to ASC BMI better than a 2D co-culture system. Primary mammary adipose stromal cells derived from obese patients exhibited increased aromatase mRNA compared to lean controls	Multiple ASC donors used	[32]
**Spheroids**	ASCs, MDA-MB-231, MCF-7, DT28, and HMLER3 CSC	Yes	Yes	Yes	The proportion of mammosphere-forming cells and cells expressing stem-like markers increases when in direct or indirect contact with adipocytes	Multiple ASC donors used	[62]
	Multipotent adipose-derived stem cells (MADS)-adipocytes, breast adipocytes, MCF-7, and MDA-MB-231	Yes	Yes	No	Adipocyte lipid droplet size decreases in the presence of mammospheres. UCP1 expression is dependent on adipocyte-mammosphere distance. Mammospheres produce adrenomedullin, which is critical in the interactions between adipocytes and breast cancer cells	hMADS cells isolated from young donors [63]	[64]
	ASCs, MDA_MB-231, and MCF-7	Yes	No	No	ASC C-C motif chemokine ligand 5 (CCL5) expression was elevated when co-cultured with the MDA-MB-231 TNBC cell line	One ASC donor used	[65]
	3T3-L1 pre-adipocytes, SKBR-3, MDA-MB-231, and MDA-MB-468	Yes	No	No	Adipocytes increase the invasiveness of breast cancer cells	Used 3T3-L1 preadipocytes	[43]
	ASCs, MCF10AT, MCF10A, MCF10DCIS.com, MCF10CA1a	Yes	Yes	No	ASCs promote premalignant breast cell invasions via direct cell contact. Obese ASCs have a pro-invasive effect on premalignant and malignant breast cell lines	Combination of models established in study	[66]
**Bioprinting**	ASCs and MDA-MB-231	Yes	No	No	MDA-MB-231 TNBC cell line induces adipose tissue ECM remodeling and lipid content modulation	Used hyaluronic acid-based bioink and extrusion-based bioprinting	[67]
	ASCs and MDA-MB-231	Yes	Yes	Yes	Adipose cells hasten the invasion and escape of tumor cells via soluble factor secretion. Tumor invasion and escape are more strongly induced by ASCs than adipocytes	Multiple demographics represented in ASC donor selection	[31]

#### 1.2.2. Spheroids 

Although three-dimensional scaffold-based platforms provide support for cell attachment and support cell proliferation, differentiation, and ECM deposition, some scaffolds can limit direct cell–cell interaction [68]. Unlike 2D and scaffold-based cultures, spheroids cultured in a scaffold-free system take advantage of the inherent self-assembly tendency displayed by multiple cell types [69]. Instead of relying on the addition of foreign ECM, this model relies on the direct generation of ECM from the cells themselves. Spheroids have already achieved success in breast cancer research as biomimetic in vitro models to study underlying mechanisms in tumor biology (Table 1) [2,21,62,70,71,72,73,74,75]. Although the exclusion of stromal cell populations such as adipocytes or ASCs in spheroid models is a current shortcoming, progress has been made in this model, as demonstrated by the promising results from several studies [49,62,64,65,66]. The spheroid models highlighted in this section focus on the adipocyte or ASC-breast cancer crosstalk. Picon-Ruiz et al. generated mammospheres composed of malignant mammary epithelial cells (MDA-MB-231, MCF-7, and T-47D) in direct contact with immature adipocytes or in the presence of the adipocyte secretome [62]. Findings suggest that mammosphere-forming cells and cells expressing stem-like markers proliferate in the presence of adipocytes or the adipocyte-derived secretome. Pare et al. demonstrated that mammosphere-secreted adrenomedullin (ADM) is critical in the control of adipocyte-breast cancer cell interactions and could potentially be interrogated for targeted therapy [64]. ASC paracrine activity in the release of CCL5 has also received attention in a co-cultured spheroid model. Results indicated an upregulation in CCL5 and C-C chemokine receptor 1 (CCR1) expression in ASC/MDA-MB-231 spheroids, further validating the pro-metastatic role of adipose tissue [65].

Spheroids can also be cultured within a scaffold system. In contrast to the models described above, He et al. created spheroids composed of breast cancer cells and 3T3-L1 adipocytes embedded in Matrigel to evaluate MDA-MB-231 and MDA-MB-468 invasiveness [43]. Similarly, Ling et al. created multicellular spheroids embedded in collagen composed of either B6.Cg-Lepob/J (ob/ob) or wild-type C57BL/6j-derived ASCs and premalignant or malignant breast cell lines [66]. In contrast to other spheroid models, Ling et al. directed special attention to the effect of the obesity-associated myofibroblastic ASC phenotype on the migration and invasion of premalignant breast cell lines. Findings of this study suggested that obese ASCs promoted tumor cell invasion in a cell contact-dependent manner more than lean ASCs. Importantly, while some of the studies included patient-derived adipocytes or ASCs [49,62,64], others included murine-derived 3T3-L1 preadipocytes or freshly isolated murine-derived ASCs [43,65,66]. 

#### 1.2.3. Microfluidics

Microfluidic technology, a subset of what is referred to as organ-on-a-chip technology, introduces a shift from a static to a more fluidly dynamic environment in which tumor models can respond to cues including fluidic pressure and flow. Microfluidic devices also replicate the in vivo vasculature and enable studies to evaluate metastasis and tumor cell migration, invasion, and adhesion from a primary to a distant tumor site [76]. Attrition of commonly used cancer treatments such as cisplatin has been evaluated using microfluidic technology, such as the Nortis Bio dual-channel microphysiologic chip [77]. Microfluidic technology can support both direct and indirect co-cultures. In indirect co-culture, single or co-cultured cell types are separated by a physical barrier so that communication can only transpire via secreted factors. They can also incorporate both two-dimensional and three-dimensional formats in which cells are either cultured directly on devices or fully encapsulated into the desired ECM [35]. For example, select models have evaluated the efficacy of doxorubicin delivery into breast tumor spheroids by mimicking vascular flow, malignant epithelial cell invasion into healthy stroma, and breast ductal carcinoma in situ (DCIS) progression to invasive ductal carcinoma (IDC) [2,78,79,80]. Despite advancements in organ-on-a-chip technology, few studies have demonstrated the utility of examining adipose-breast cancer interactions in a comprehensive manner. Rahman et al. and Morgan et al. assessed the effect of human ASCs on breast cancer cell responses to chemotherapy (Taxol) or aromatase inhibitors (anastrozole) [30,32]. While Rahman et al. focused on the impact of a single ASC donor on MDA-MB-231 morphology and treatment response, Morgan et al. examined MCF-7 anastrozole sensitivity with respect to BMI and included lean (mean BMI 24 kg/m^2^) and obese (mean BMI 34 kg/m^2^) patient-derived ASCs for comparison. 

#### 1.2.4. Bioprinting

Despite its relevance and impact in driving disease progression, recapitulating the complex microarchitecture of breast TME has proven to be an arduous task. However, with advancements in 3D bioprinting (3DBP) technology, more customizable, comprehensive models have been developed, including systems mimicking vascular-like tubes, artificial skin, lung, kidney, cartilage, and brain [35]. Bioprinted models simulating breast, brain, ovarian, and skin cancers have been optimized over the past five years [2,81,82,83,84,85]. Bioprinting can be described as a process that deposits materials by layering to generate a three-dimensional structure. Current commonly used bioprinting materials include alginate, decellularized ECM, and microcarriers or hydrogels. These materials are assembled into tissue structures such as spheroids, cell pellets, or organoids [35]. Dance et al. engineered micropatterned type I collagen gels into human breast tumors adjacent to a stroma composed of adipocytes, ASCs, and lymphatic-like cavities to model the interstitial invasion and vascular escape of breast cancer cells (Table 1) [31]. This model demonstrates that tumor escape and invasion are strongly supported by ASCs, more so than adipocytes [31]. In contrast, Horder et al. generated ASC spheroids encapsulated in a hyaluronic acid-based bioink via extrusion-based bioprinting (EBB) (Table 1) [67]. After adipogenic differentiation, the adipose microtissue was merged with a bioprinted breast tumor compartment to form an adipose-breast cancer tissue construct to assess tumor-induced ECM remodeling in conjunction with lipid modulation [67].

In the development of breast cancer MPS models for preclinical studies, bioprinting provides the inclusion of multiple cell types in the models, generating complex and physiologically relevant tissue structures. Furthermore, automation for MPS with this technology supports optimization to support efficient and reproducible fabrication processes [86,87].

#### 1.2.5. Xenograft and PDX Models 

Breast cancer PDX models can offer predictive power compared with long-established cell lines and transgenic murine models [88]. PDX models are characterized by the implantation of patient-derived tumor tissue into immunocompromised mice [60]. PDX models for cancer studies typically utilize humanized mouse models to mimic the human immune system. Unlike cell-line-derived xenograft models, PDX models permit the retention of patient tumor heterogeneity, mutations, TME, and endocrine function, making them an ideal model for the evaluation of biomarkers and cell-based therapies, preclinical studies, and personalized medicine approaches [18,89]. 

In contrast to PDX models, traditional orthotopic xenograft models utilize immortalized cell lines, which arguably lack tumor heterogeneity and retention of tumor tissue architecture and stroma. Although great strides have been made in demonstrating the feasibility of PDX models, as they have exhibited responses parallel to tumor responses in vivo, there are still gaps in knowledge concerning adipose tissue-breast cancer crosstalk with respect to PDX models [90]. Sabol et al. conducted a side-by-side comparison of orthotopic xenograft and PDX models using established breast cancer cell lines or PDX in combination with pooled ASCs that were isolated from lean (BMI < 25 kg/m^2^) or obese (BMI > 30 kg/m^2^) adipose tissue donors (Table 2) [91]. Results from this study demonstrated the impact of the ASC secretome on breast cancer cell proliferation and phenotype, including obesity-affected EMT of TNBC cells. Sabol et al. also established a xenograft model in which MCF-7 cells co-cultured with ASCs from an obese donor were exposed to radiation prior to injection (Table 2) [49]. The study illustrated the impactful nature of adipose tissue dysfunction and obesity on breast cancer treatment responses.

The role of adipose–epithelial cell interaction was highlighted by Goto et al. as they interrogated the effect of adipocyte-secreted adipsin (also known as complement factor D) on breast cancer cell proliferation and the adoption of a stem cell-like phenotype via 3D culture and xenotransplantation assays (Table 2) [92]. By employing the co-transplantation of ASCs or adipsin knockdown ASCs (shAdipsin) and breast cancer PDX cells, Goto et al. demonstrated that adipsin effectively enhanced the growth of PDX tumors in vivo, and thus could serve as a potential target for adipsin/C3a inhibitors such as lampalizumab.

The distinct and heterogeneous nature of PDX models is exemplified in the study that was conducted by Matossian et al., in which a new PDX model for metaplastic breast cancer (MBC) was extensively characterized and validated as a translatable platform for the development of novel therapies (Table 2) [60]. Of particular interest was the presence of a cancer stem cell (CSC)-like population within the TU-BcX-41C PDX model. To evaluate the maintenance of this population, Matossian et al. modified a previously published system that was suggested to be both translatable and physiologically relevant to in vitro TME [60,61,93]. The modified system, identified as the breast cancer microphyisological system (BC-MPS), was composed of human-derived breast adipose tissue, ASCs from two separate obese patients, and the Tu-BcX-41C cell line. Their results revealed that there was an elevated expression of the CSC marker, GD2 ganglioside, in the BC-MPS compared to traditional 2D culture conditions and further validated the powerful nature of three-dimensional in vitro culture.

**Table 2:** Review of in vivo breast cancer-adipose preclinical platforms for breast cancer studies, which includes a summary of associated cell types, derivation, and key findings demonstrated in each study.

Platform	Cell Type(s)	Human-Derived (Y/N)	Patient-Derived Adipocytes or ASCs (Y/N)	Multi Patient Adipocytes or ASCs (Y/N)	Key Findings	Reference
**Xenograft**	ASCs, BT20, MDA-MB-231, MDA-MB-468, MCF-7, and HCC1806	Yes	Yes	No	ASCs derived from obese donors promote a pro-metastatic phenotype by upregulating epithelial–mesenchymal transition (EMT)-associated genes and promoting migration in vitro	[91]
	ASCs, MCF-7, T47D, and ZR-75	Yes	Yes	Yes	MCF-7 co-cultured with obese ASCs and irradiated prior to injection had increased tumor growth compared to cells that were not co-cultured before radiation	[49]
**PDX**	MDA-MB-231, TU-BCX-41C PDX, and TU-BCX-41C PDX derived cells	Yes	No	No	Provided a detailed characterization of a PDX model for metastatic breast cancer (MBC). The established PDX model maintained consistent matrix architecture and stiffness after multiple serial passages	[60]
	ASCs, human breast cancer PDX cells	Yes	Yes	Yes	Adipsin secreted from mammary ASCs promotes cancer stem cell-like properties and proliferation of human breast cancer PDX cells in vitro and in vivo	[92]
	TU-BCX-2 K1 PDX, ASCs	Yes	Yes	Yes	ASCs derived from obese donors promote a pro-metastatic phenotype by upregulating EMT-associated genes and promoting migration in vitro	[91]

## 2. Discussion

The adoption of new, innovative approaches to evaluate breast cancer-adipose tissue crosstalk with the inclusion of more traditional methods has facilitated the development of novel clinically relevant, translatable preclinical models. Although considerable progress has been made, there remains room for improvement. With preclinical model innovation, emphasis lies on the feasible application of precision medicine and the ability to tailor medical treatment to the individual characteristics of each patient. One of the overarching goals of precision medicine is to classify individuals into subpopulations that differ in their susceptibility to a particular disease or treatment response [94]. This will facilitate better healthcare decisions, effective treatment options, and the quality of care initiated. 

Many models, including in vitro cell culture, cell-line or patient-derived xenografts, or murine/nonmurine animals, are subpar in complexity and void of consideration and/or incorporation of a TME and ECM [95]. With that said, focus must be directed on the impact of intra-tumoral heterogeneity on patient prognosis, survival, and treatment efficacy. One of the most recognized examples of intra-tumoral heterogeneity is within the TNBC population. Gene expression analyses have identified several molecular subtypes with distinct mutational profiles, genomic alterations, and biological processes, including basal-like (BL), immunomodulatory (IM), luminal androgen receptor (LAR), mesenchymal (M), and mesenchymal stem-like (MSL) [96]. Preclinical models not only reflect intra-tumoral heterogeneity and account for variance in the landscape of the microenvironment but must also mirror the intra-tumoral heterogeneity observed amongst patients. Many factors play a critical role in the onset and progression of breast cancer, including genetic background [97]. Specific population subsets within the population at large have a genetic predisposition to diseases. Some examples include the Ashkenazi and Tay Sachs, Caucasians and Cystic Fibrosis, and African Americans and Sickle Cell Disease [98,99,100]. 

Similarly, variances with respect to patient demographics such as race, ethnicity, and age have been recognized as key players in the development of preclinical breast cancer models for drug efficacy [8,101,102,103]. To compound the demographic-related challenges, the functional role of adipocytes and adipose tissue must also be taken into careful consideration when integrating the adipose stromal environment into preclinical models [22]. With respect to experimental design, we must assume that (a) adult stem cell function is dependent on patient or donor physiology and the anatomical harvest location [15], and (b) the heterogeneity of the resident cell populations in adipose tissue varies from patient to patient and donor to donor [15]. 

Several of the preclinical models included in this review continue to incorporate murine-derived cells, or ECM, versus human-derived cells, or ECM. Although murine-derived ECM such as Matrigel is valuable and feasible in establishing an MPS model, the use of these types of matrices does not provide an accurate model of the human disease, largely due to their origin, batch-to-batch variability in gelation, and biomechanical properties [104,105]. In addition, mouse adipose tissue, cellular composition, and corresponding metabolic signaling pathways are strikingly different from human adipose tissue, which has been demonstrated to diminish preclinical model translatability and predictability [106]. Furthermore, studies that include human-derived ASCs or adipocytes incorporate a single donor, either with or within corresponding demographics. 

In this review, we highlighted that 60% of the in vivo platforms and 38% of the in vitro platforms not only incorporated human-derived ASCs or adipocytes and patient-derived ASCs or adipocytes but also included a range of donors that varied in age, race, ethnicity, and BMI. Of the in vitro platforms described in this article, <10% included the ASC or adipocyte donor demographics. Although 100% of this small percentage disclosed donor BMI, only <50% included donor age, and 1% utilized non-white ASCs in their study. The inclusion of both immune and endothelial cells into adipose-breast cancer models would further improve their translatability and more accurately mimic the in vivo tumor microenvironment. Only one of the preclinical models reviewed included immune cells. Importantly, recent studies have demonstrated strong depot-specific associations and BMI-dependent shifts in resident cell populations, including adipocytes, ASCs, vascular, and immune cells, within different adipose depots [107]. The incorporation of human SVF cells into current preclinical models would help mitigate some of these variances. The SVF, consisting of a heterogeneous population of cells including ASCs, hematopoietic stem and progenitor cells, endothelial cells, erythrocytes, fibroblasts, lymphocytes, monocytes/macrophages, and pericytes, has more utility, ease of use, and patient-associated heterogeneity than the adipocyte and/or ASC subpopulations alone [108]. Improving breast cancer-adipose tissue preclinical model diversity requires several directives, including: (1) acquiring a diverse donor pool of matched mammary adipose tissue and breast tumors; (2) characterizing the breast TME of a diverse population with special attention to age, race/ethnicity, and other comorbidities; and (3) improving the cellular heterogeneity of the models by incorporating endothelial and immune cell populations. Initial steps to improve the diversity of these models could include the use of ASC donor pools for the preliminary study, followed by studies using single ASC donor samples to define interindividual differences in drug responses. 

Companion technologies developed over the past several years have provided additional value for microphysiological systems in preclinical studies as well as for the understanding of breast cancer initiation, progression, and invasion. The application of high-content imaging techniques allows investigators to extract information within multicellular 3D environments. This, in combination with computer-assisted data analysis and machine learning, will enable high-throughput modeling and application of behavior and responses within the microphysiological systems. Indeed, high-throughput, label-free, live cell imaging of MPS offers the potential to become a foundational tool in future pharmaceutical drug discovery [109]. In addition, the incorporation of biosensors for readouts in real-time [110,111], as well as the connectors and flow generators in the plates and devices [112,113,114], will support the adoption of breast cancer MPS alone or in multi-tissue systems for drug toxicity and efficacy testing in the context of cancer [115,116,117]. 

## 3. Conclusions

Technical innovation and inclusion of patient diversity in the experimental designs are slowly improving the development of physiologically and genetically relevant preclinical models and will be critical elements for capturing the key aspects of the breast TME as they relate to each racial and ethnic subpopulation [23]. The use of microphysiological models using human-derived materials as well as historical data from preclinical models and clinical trials allows for comparative studies and validation of breast cancer MPS. This can confirm target validity with established clinical targets as well as prediction validity, in which clinically effective agents can better support the identification of future novel agents [23]. Better models will provide a more in-depth understanding of breast cancer pathophysiology and the development of targeted treatments for each breast cancer subgroup in the broader human population. Despite the growth of the MPS industry over the past two decades, these systems have not yet gained overwhelming traction in the pharmaceutical sector, largely in part due to the lack of supportive datasets and the time and monetary investments required by pharmaceutical companies to assess the overall value of the technology [118]. Verification and validation of MPS for safety and efficacy of anticancer drugs required from the U.S. Food and Drug Administration (FDA) in clinical trials are not as defined as for animal studies [119]. Guidelines for the verification/validation of MPS will be critical to standardizing the systems now that MPS has been cleared to replace animal research in preclinical studies. Validation of MPS models would require comparative studies using the current gold standard animal models for specific readouts. Furthermore, the standardization and optimization of MPS models could include an intermediate step using an MPS model composed of animal-derived tissues that would be compared to animal studies in terms of cellular and molecular responses. Ultimately, an accurate, reliable, and relevant model for drug screening could serve as a personalized medicine tool for evaluating and addressing treatment resistance, for example, in a targeted manner for at-risk ethnic subpopulations or at the individual patient level.
